# New Books

**Published:** 2009-04

**Agenda for a Sustainable America**

John Dernbach

Washington, DC:Island Press, 2009. 520 pp. ISBN: 978-1-58576-133-3, $52.95

**Bioinformatics: A Concept-Based Introduction**

Venkatarajan Mathura, Pandjassarame Kangueane

New York:Springer, 2009. 205 pp. ISBN: 978-0-387-84869-3, $69.95

**Clouds in the Perturbed Climate System: Their Relationship to Energy Balance, Atmospheric Dynamics, and Precipitation**

Jost Heintzenberg, Robert J. Charlson, eds.

Cambridge, MA:MIT Press, 2009. 576 pp. ISBN: 978-0-262-01287-4, $40

**Contaminated Sediments**

Tarek A. Kassim, Damià Barceló, eds.

New York:Springer, 2009. 181 pp. ISBN: 978-3-540-88013-4, $189

**Coping with Climate Change: National Summit Proceedings**

Rosina M. Bierbaum, Dan Brown, Jan McAlpine, eds.

Washington, DC:Island Press, 2008. 256 pp. ISBN: 978-1-59726-556-0, $29.50

**Energy and Environmental Challenges to Security**

Stephen Stec, Besnik Baraj, eds.

New York:Springer, 2009. 456 pp. ISBN: 978-1-4020-9452-1, $119

**Energy Studies – Problems and Solutions**

W. Shepherd, D.W. Shepherd

Hackensack, NJ:World Scientific Publishing Co., 2008. 292 pp. ISBN: 978-1-84816-405-5, $88

**Environmental Justice and Sustainability in the Former Soviet Union**

Julian Agyeman, Yelena Ogneva-Himmelberger, eds.

Cambridge, MA:MIT Press, 2009. 320 pp. ISBN: 978-0-262-51233-6, $25

**Influence of Climate Change on the Changing Arctic and Sub-Arctic Conditions**

Jacques C.J. Nihoul, Andrey G. Kostianoy, eds.

New York:Springer, 2009. 232 pp. ISBN: 978-1-4020-9458-3, $219

**Marine Toxins as Research Tools**

N. Fusetani, W. Kern, eds.

New York:Springer, 2009. 259 pp. ISBN: 978-3-540-87892-6, $199

**New Developments in Biostatistics and Bioinformatics**

Jianqing Fan, Xihong Lin, Jun S. Liu, eds.

Hackensack, NJ:World Scientific Publishing Co., 2009. 300 pp. ISBN: 978-981-283-743-1, $78

**Restructuring Federal Climate Research to Meet the Challenges of Climate Change**

National Research Council, Committee on Strategic Advice on the U.S. Climate Change Science Program

Washington, DC:National Academies Press, 2009. 178 pp. ISBN: 978-0-309-13173-5, $41

**Science and Decisions: Advancing Risk Assessment**

National Research Council, Committee on Improving Risk Analysis Approaches Used by the U.S. EPA

Washington, DC:National Academies Press, 2009. 424 pp. ISBN: 978-0-309-12046-3, $64.50

***Science*** **Magazine’s State of the Planet 2008–2009**

Donald Kennedy, ed.

Washington, DC:Island Press, 2008. 216 pp. ISBN: 978-1-59726-406-8, $19.96

**Structuring an Energy Technology Revolution**

Charles Weiss, William B. Bonvillian

Cambridge, MA:MIT Press, 2009. 280 pp. ISBN: 978-0-262-01294-2, $24

**The Design of Climate Policy**

Roger Guesnerie, Henry Tulkens, eds.

Cambridge, MA:MIT Press, 2009. 408 pp. ISBN: 978-0-262-07302-8, $38

**The Landscape of Reform: Civic Pragmatism and Environmental Thought in America**

Ben A. Minteer

Cambridge, MA:MIT Press, 2009. 280 pp. ISBN: 978-0-262-51255-8, $15

**The World’s Water 2008–2009: The Biennial Report on Freshwater Resources**

Peter Gleick

Washington, DC:Island Press, 2008. 432 pp. ISBN: 978-1-59726-505-8, $35

**Toward Sustainable Communities, 2nd ed.**

Daniel A. Mazmanian, Michael E. Kraft, eds.

Cambridge, MA:MIT Press, 2009. 352 pp. ISBN: 978-0-262-13492-7, $50

